# 2278: Identifying causative mutations in Treacher Collins syndrome using iobio

**DOI:** 10.1017/cts.2017.64

**Published:** 2018-05-10

**Authors:** Alistair N. Ward, Matt Velinder, Chase Miller, Tony Di Sera, Yi Qiao, Dave Viskochil, Gabor Marth

**Affiliations:** The University of Utah School of Medicine, Salt Lake City, UT, USA

## Abstract

OBJECTIVES/SPECIFIC AIMS: The objective of the study was 2-fold; to identify potentially deleterious alleles in a child with Treacher Collins syndrome, and; to demonstrate the value of the iobio analysis platform for intuitively and rapidly analyzing genomic data. METHODS/STUDY POPULATION: We used the iobio suite of web-based applications to analyze quality metrics for the sequencing data and called variants for the proband and his parents. We then visually interrogated variants in genes potentially associated with the syndrome in real-time, using the intuitive gene.iobio application. We sought high impact variants that demonstrated a predicted impact on the protein function, and were simultaneously at low allele frequency in the general human population. Variants were also compared against the ClinVar database of known mutations to identify variants that have already been associated with this, or related syndromes in the literature or clinical studies. Finally, the gene.iobio tool allows users to interrogate the primary sequencing data to ensure that no variants had been missed by the primary variant calling pipeline. This analysis pipeline was performed using intuitive web-based apps in real time, and consequently represents a system that is available to users that traditionally are excluded from these analyses. RESULTS/ANTICIPATED RESULTS: The iobio suite was used to rapidly assess data quality and interrogate genetic variants for a child with Treacher Collins syndrome. A compound heterozygote consisting of 2 missense alleles in the TCOF1 gene was identified as a compelling pathogenic allele, necessitating further functional investigation. The study helped validate the use of the intuitive iobio tools in such analyses, strengthening the case for greater involvement of medical professionals in data analysis. DISCUSSION/SIGNIFICANCE OF IMPACT: The performed analyses demonstrated that the whole genome sequencing data for the family being studied was of a very high quality, although 1 gene demonstrated a local region of almost zero coverage. This ensured that study conclusions can be presented with confidence. A variant associated with Treacher Collins syndrome 1 in ClinVar was uncovered in the TCOF1 gene, however, given it’s benign rating, this variant was not considered further. The most interesting candidate was a compound heterozygote, consisting of 2 missense mutations, also in the TCOF1 gene. These mutations occurred with allele frequencies of 22% and 8% in the general population, and additional molecular and functional studies are currently being pursued.

